# Effects of nordic walking exercise on gait, motor/non-motor symptoms, and serum brain-derived neurotrophic factor in individuals with Parkinson's disease

**DOI:** 10.3389/fresc.2022.1010097

**Published:** 2022-10-14

**Authors:** Cathy C. Harro, Michael J Shoemaker, Cassandra M. Coatney, Valerie E. Lentine, Lillian R. Lieffers, Jessica J. Quigley, Shannon G. Rollins, Jonathan D. Stewart, Julie Hall, Sok Kean Khoo

**Affiliations:** ^1^ Department of Physical Therapy and Athletic Training, Grand Valley State University, Grand Rapids, MI, United States; ^2^ Department of Medical Laboratory Science, Grand Valley State University, Grand Rapids, MI, United States; ^3^ Department of Cell and Molecular Biology, Grand Valley State University, Grand Rapids, MI, United States

**Keywords:** Parkinson's disease, nordic walking, exercise training, gait function, exercise biomarkers

## Abstract

**Objective:**

The primary purpose of this study was to investigate the immediate and long-term effects of Nordic Walking (NW) exercise on walking function, motor/non-motor Parkinson's Disease (PD) symptoms, and serum brain-derived neurotrophic factor (BDNF) in persons with idiopathic PD.

**Methods:**

Twelve community-dwelling participants with mild to moderate idiopathic PD and varied degrees of gait dysfunction were recruited for this prospective, repeated measures design that examined clinical measures and BDNF levels at baseline (T0), post-intervention (T1) and 3-month follow-up (T2). Participants engaged in 6 weeks of supervised NW exercise training with individualized instruction, followed by 14 weeks of independent NW exercise with remote coaching. Outcome measurements included daily step counts, 6-Minute Walk Test (6-MinWT), 10-Meter Walk Test (10MWT), spatiotemporalparameters, Timed Up and Go Test (TUG), dual-task TUG, Revised-Movement Disorder Society-Unified Parkinson's Disease Rating Scale (MDS-UPDRS), Revised-Freezing of Gait Questionnaire, MDS-Nonmotor Symptom scale (NMS), Parkinson's Fatigue Scale, and serum BDNF levels. The Friedman test with *post hoc* Wilcoxon sign-ranked pairwise comparisons were used to compare baseline to T1, baseline to T2, and T1 to T2 timepoints with a Benjamini-Hockberg correction applied.

**Results:**

Statistically significant improvements found post-training and retained at 3-month follow-up included 6-MinWT, daily step count, 10mWT, MDS-UPDRS, and TUG with effect sizes of 0.57 to 1.03. Serum BDNF at T2 was significantly greater than T0 and T1. Although no statistically significant improvements were observed in the MDS-NMS, 9 of 12 participants had improved non-motor symptoms. There was good adherence, sustained independent exercise engagement, and no adverse events over the 5-month study duration.

**Conclusions:**

This study demonstrated that NW exercise was a safe, feasible, and sustainable mode of aerobic exercise for this sample of participants with varied Parkinson's disease duration and severity. Following an individualized and progressive NW training intervention, significant improvements in walking function, daily activity level, and motor function were observed. Following the supervised NW training phase, independent three-month engagement in NW exercise was sustained with long-term retention of these clinical improvements and an increase in serum BDNF levels over this five-month NW exercise trial.

**Impact:**

Nordic walking exercise may be a safe, feasible and sustainable mode of independent exercise for improving daily ambulatory activity, gait and motor function, and serum BDNF in individuals with mild to moderate PD with varied gait abilities.

**Clinical Trials Registry ID:**

20-101-H

## Introduction

Parkinson's disease (PD) is a highly prevalent neurodegenerative disorder in older adults that leads to reduced activity levels, physical disability, and accelerated age-related decline in mobility due in part to progressive gait dysfunction and postural instability (1–6). A recent meta-analysis supports that compared with age-matched healthy controls, individuals with PD demonstrate reduced self-selected gait speed, stride length, cadence, and sagittal hip angle, as well as altered double support and swing time (7). Prevention of balance, gait, and functional decline through early, long-term physical activity-and exercise-based interventions are priority goals in physical therapy management for individuals with PD (8–12). The underlying mechanisms by which this exercise-based intervention may effect changes in PD need to be further elucidated (9, 10).

Low physical activity and ambulatory activity are reported in those with PD including significantly less sustained bouts of walking and a decline in step counts by nearly 12% per year (13, 14). This reduced activity level is an early risk factor for possible accelerated gait and functional decline. As walking function declines, the incidence of falls significantly increases, with fall risk 5 times higher in those with PD compared to healthy age-matched peers (15, 16). Freezing of gait is experienced in more than 50% of individuals with PD, which also markedly contributes to gait disability and increased fall risk (17–19).

Sustained engagement in moderate physical exercise and higher activity levels (e.g., > 150 min per week) is associated with a preservation of walking function in PD (12, 20, 21) and appears to be neuroprotective for slowing disease progression (10, 21, 22). Consistent scientific evidence supporting the neuroprotective effects of moderate to high intensity exercise in both clinical and animal studies have shown beneficial cellular changes including: (1) increased blood flow and angiogenesis, (2) up-regulated neurotrophic factors, (3) reduced neuroinflammation, and (4) enhanced immune responses (9, 22–26). Brain-derived neurotropic factor (BDNF) is an important neurotrophin that promotes neuron survival, differentiation and neurotransmission. It is essential to keep the dopaminergic neurons intact, protect them from apoptosis, and sustain neuroplasticity. In PD, BDNF expression in the brain and serum are significantly reduced when compared to healthy controls (27–29). Studies in both animals and humans have shown that BDNF levels increase after exercise, including in persons with PD (30–34).

Individualized aerobic exercise prescription that is task-specific, challenging, and feasible for an independent exercise program is needed for individuals with PD to optimize their walking and motor function and sustain long-term engagement in regular physical exercise (12). Walking as a form of aerobic exercise has been reported to be safe and to improve functional and gait outcomes and quality of life in PD (35). Although treadmill training is supported in research as effective for improving cardiovascular and walking function in PD (36–38), this intervention often requires supervised training and access to equipment. Nordic walking (NW) is a form of fitness walking using specialty poles that mimics the full body movement pattern of cross-country skiing and can be performed in varied terrains (39). Energy expenditure during NW in healthy adults generates 6.3–7.7 METS (metabolic equivalent of task) as compared to 3.3–5.0 METS walking over ground without poles (40, 41). In addition to the documented aerobic conditioning effects in older adults (41–43), NW has targeted benefits that may address PD-related gait dysfunction and ameliorate gait decline, including rhythmic timing of interlimb coordination, increased engagement of upper extremities and trunk (44, 45), and promotion of improved stride length, arm swing, gait speed and walking distance (44, 46–48). As an intervention stimulus, NW increases external mechanical work and the pendular mechanism of gait (49). Additionally, the NW pattern facilitates upright trunk posture, increased medio-lateral margin of stability and greater trunk coordinative stability (50, 51), which enhances postural stability when walking with the poles, potentially reducing fear of falling (52–54). Additionally, the use of poles may serve as an external cue to help with gait rhythm and automaticity, similar to rhythmic auditory cued gait training (47, 55).

Clinical research examining the therapeutic effects of NW in persons with PD is limited and is in its early stages. A systematic review by Bombieri et al (44). pooled data from 4 randomized clinical trials for meta-analysis and found that NW was statistically better than exercise controls for improving PD-motor symptoms as measured by the Movement Disorder Society-Revised Unified Parkinson Disease Rating Scale (MDS-UPDRS) motor score. A second systematic review (40) reported that in four of six randomized clinical trials NW resulted in greater gains in functional gait measures and UPDRS-motor scores than active controls, including treadmill training, overground walking, and conventional exercise groups (46–48, 54, 56). Few studies have investigated the effects on NW exercise on non-motor PD symptoms with preliminary support for improved cognition, attention, and apathy (47, 54). Most recently, Passos-Monteiro et al. demonstrated that NW exercise resulted in significant improvements in cognitive function, depressive symptoms, and physical and psychological aspects of quality of life in persons with PD (57).

Despite the favorable preliminary research results of NW in persons with PD, several gaps remain. First, dosing of NW intervention and exercise prescription is highly variable across studies. Cugusi et al. (40) noted that the heterogeneity in NW methods across studies made it difficult to draw firm conclusions. Second, the majority of these clinical studies examined only the immediate effects of NW exercise programs and did not assess retention or long-term outcomes. Third, research that investigates long-term carryover of independent NW exercise following a supervised training program is lacking. Finally, it is not known which modes of exercise training have positive effects on biomarkers that are indicative of biochemical changes to support PD brain health. Therefore, the overall premise of this proof-of-concept pilot study is that this intensive fitness NW may produce changes at the cellular and molecular levels. Investigating changes in walking and motor/non-motor function following NW exercise and associating these changes with exercise-related biomarkers may provide foundational support for the neuroprotective benefit of NW in persons with PD.

The specific aims of the present paper are to: (1) investigate the immediate and long-term effects of NW exercise on walking function, motor/non-motor PD symptoms, and BDNF levels in persons with mild to moderate idiopathic PD, (2) determine the feasibility of implementing an individualized, progressive NW exercise program, and (3) examine independent NW exercise engagement after a supervised training program to assess feasibility and sustainability of this mode of task-specific aerobic exercise.

## Methods

### Study design

This prospective, repeated measures study included a 3-week baseline with two time points for assessment of dependent measures (T0-A and T0-B) followed by a 6-week NW training intervention phase with an immediate post-training assessment (T1), and a 3-month independent NW exercise phase with a follow-up assessment at the end of the 3 months (T2). Prior work demonstrated significant improvements in walking and balance measures following a 6-week treadmill and rhythmic over ground auditory cueing protocol (58) and in a 6-week NW case series (59), and therefore, it was expected that a 6-week training session would be sufficient to induce training effects_._ NW exercise adherence during both the supervised and independent phases, as well as any adverse effects were monitored to determine feasibility and safety, particularly for the independent NW phase of the study.

### Participants

Ambulatory, community-dwelling individuals with idiopathic PD were recruited from local Parkinson's support groups, community-based PD exercise classes, and local physical therapists. A two-stage screening process using an initial phone screen followed by an in-person screen was employed to assess if individuals qualified for the study based on the following criteria. Inclusion criteria were: (1) individuals with mild to moderate disease severity (Modified Hoehn and Yahr stage I-III), able to safely and continuously ambulate a minimum distance of 500 feet independently without an assistive device and ascend/descend a full flight of stairs with or without the use of railings with no more than standby assistance, (2) have functional vision with or without corrective lenses for safe outdoor mobility, and (3) stable Parkinson's medications and dosing over the past month. Exclusion criteria were: (1) no other neurologic diagnoses (such as brain injury or stroke), (2) no significant comorbidities (cardiorespiratory conditions, chronic lung disease, orthopedic conditions, or recent orthopedic surgery) that would limit their ability to safely participate in an intensive walking exercise program, (3) had significant cognitive impairment as determined by a Montreal Cognitive Assessment score below 21 points (60), (4) no recent deep brain stimulation (within the last 3 months) or planned in the next 4 months, and (5) had previously been trained in NW technique and was currently engaged in moderate intensity NW exercise at least 3 days per week. All participants provided informed consent. The protocol was approved by the Grand Valley State University Institutional Review Board and was registered with ClinicalTrials.gov. (Protocol #20-101-H).

### NW intervention

#### Intervention procedures

Nordic Walking training protocol consisted of a total of 14 one-hour sessions over a 6-week period, training three times per week for the first two weeks and twice weekly for the last four weeks. For the supervised training, the coaches provided one-on-one individualized NW training. The independent training phase consisted of fourteen weeks of independent NW exercise three times per week, with coaches contacting participants twice per month to assess progress and promote continued engagement. During the NW intervention period, participants were asked to abstain from engaging in direct physical therapy services that involved therapeutic gait training or treadmill walking, or if physical therapy services were warranted to notify researchers. Participants were fitted with customized Nordic walking poles (SWIX Nordic Ski Walking VIP poles; Swix Sport USA, Inc. 60 Newark Street, Haverhill, MA, 01832) and issued a physical activity tracker, *Fitbit Inspire HR*™ (405 Howard St, San Francisco, CA 94105), which has been shown to monitor physical activity with good adherence and accuracy in persons with mild to moderate PD (61–63). This device allowed researchers to monitor heart rate during NW training as well as provided data on training volume and daily steps.

#### Supervised training phase

The supervised NW training phase was administered by the primary investigator (PI) who is a Board-certified Neurologic Clinical Specialist and NW certified trainer, and three graduate physical therapy students who received direct education on NW technique and instruction, and coaching tips by the PI. The NW training sessions consisted of a 10-minute warm-up period using the NW poles, followed by 40–45 min of continuous NW training, and a 10-minute cool-down period. Training was conducted in a small group in an outdoor setting with a 2:1 participant to trainer ratio, which allowed for individualized instruction and progression of training. Training took place at an outdoor community track and a community park on a paved path with rolling hills or grassy terrain training options. The NW exercise program was customized for each participant by the trainers by engaging in ongoing assessment of participant's gait pattern, NW technique, and exercise responses during the training sessions. The target NW training duration goal was 45 min of continuous walking. Customized training intensity for the participants was instructed based on the use of the Borg Rating of Perceived Exertion scale (RPE) (64). The target goal for NW training intensity was 4–6/10 with intermittent high-intensity short intervals (7–8/10) during training progressions. Participants were instructed in proper NW diagonal technique, as described by International Nordic Walking Federation (39), which emphasizes upright posture, maintaining a backward pole position during the loading phase, and using poles for active push off with each step (41, 45). NW technique, gait rhythm and sequencing were facilitated with modeling, pacing, and attentional instructional cues. The NW gait characteristics that trainers focused on included increasing movement amplitude (stride length and long arm swing), increasing walking speed, more intentional engagement of upper extremities, upright posture, and gait rhythmicity. Consistent with Monteiro et al. (46) and others (51, 52, 57), NW training progression concepts that were applied included systematically increasing walking duration, speed, and intensity. In addition, progression elements included adding speed and effort intervals, skipping, and dual cognitive tasks, and NW on uneven terrain such as hills and grass (46, 48). The trainers collaboratively set weekly training goals with the participant and provided them direct feedback on NW performance and progress towards their goals. Group comradery and accountability was facilitated through small group training context and pairing individuals during training with a partner who walked at a similar gait speeds and NW skill level. Weekly supervised NW training activities and distances for each participant were documented in a training log. Additionally, participants were asked to engage in independent NW exercise at least 1–2 times weekly during the 6-week training period and complete weekly home activity logs, as well as document total daily steps based on *Fitbit* activity monitor data. Fall report data was collected weekly *via* participant interview during the Supervised Phase. Additionally, any adverse events during supervised and independent NW exercise were documented.

#### Independent training phase

Participants were asked to continue NW exercise at least three times per week independently for 3 months following the training phase. Participants documented the frequency, duration, and distance of their NW weekly exercise, any other exercise performed, and total number of daily steps in the weekly activity logs. Researchers contacted participants by phone twice monthly to promote accountability, provide remote coaching tips for their NW exercise program, and troubleshoot any potential barriers to adherence. Participants also completed a prospective monthly fall calendar to document any falls and near falls (65).

### Outcome measures

Outcome measures were administered at 2 time points over a 3-week baseline [week one (T0-A) and week three (T0-B)], immediately post-training (T1), and three months post-training (T2), except for the Movement Disorder Society- Non-Motor Scale (MDS-NMS), which was only administered at T0A, T1, and T2. Daily physical activity (steps per day) was measured between T0-A and T0-B and at T1 and T2. All measures were completed during the participants' on-phase of PD medication. Outcome measures were administered using standardized procedures and the same testing order at all time points. Researchers performing the testing were not involved in the NW exercise training component of the study, but were not blinded to study time points, except for the UPDRS-part III motor section, which was assessed by a blinded and MDS-UPDRS certified rater. The investigator scoring the UPDRS-part III motor section had no contact with subjects and upon completion of the study was presented, in random order, participants' video recordings of the motor section from each of the time points.

#### Daily physical activity

Physical activity was tracked by a *Fitbit Inspire HR*™ (405 Howard St, San Francisco, CA 94105) wrist monitor (66) worn all day every day, except for charging time. Daily steps were recorded with the intention of examining change in daily activity level over time.

#### Gait measures- 6-minWT, 10 mWT, spatiotemporalParameters

The 6-MinWT was used to assess walking endurance using standardized 100 ft. walkway and procedures from American Thoracic Society (67). The 10 mWT was used to measure gait speed at both comfortable and fast speed (m/s) conducted on measured level walkway using standardized procedures (68–70). Participants were asked to do two trials of both the comfortable and fast speed with the averages taken for each. SpatiotemporalGait Measures including Stride Length, Cadence, and Stride-to-stride Variability were assessed during comfortable and fast speeds of the 10 mWT using the BTS G-walk system (71–73) (BTS G-Walk; BTS Engineering Corp. 147 Prince St, Brooklyn, NY, 11201).

#### Functional mobility measures

The TUG, Cognitive-TUG, and Motor-TUG timed tests were used to assess mobility and dual task skill (74–76). These dual task TUG subtests were administered using standardized procedures (76). Participants were asked to complete two trials of each of the three tests at comfortable and fast speeds. The averages of each of the two trails were taken.

#### Disease severity, motor and non-motor symptoms

The MDS-UPDRS was administered by the lead researcher who is a trained and certified rater. The MDS-UPDRS is the gold standard and valid measure for assessing disease severity in PD and is commonly used to measure treatment effectiveness (77). The MDS-UPDRS has four parts: Part I (non-motor experiences of daily living), Part II (motor experiences of daily living, Part III (motor examination) and Part IV (motor complications). For this study the MDS-UPDRS total score and Part III-motor subscore were analyzed (78).

The Revised-FoG Questionnaire was administered to determine presence, frequency, severity and impact of FoG on daily mobility (79, 80). The MDS-NMS (81, 82) was used to assesses non-motor symptoms in PD. This assessment measures both the frequency and severity of 13-non-motor domains including over 52 items and covers a broad scope of non-motor symptoms. Lastly, fatigue was measured using the Parkinson's Fatigue Scale (PFS), which is the only fatigue scale developed specifically for persons with PD and has excellent test-retest reliability and construct validity (83, 84). The PFS is a 16-item patient-rated scale which was developed to assess the construct of physical fatigue and its impact on everyday function in individuals with PD.

#### Brain-derived neurotrophic factor

Blood samples were collected from 7:30 am to 9:30 am in serum clot activator tubes by venipuncture after participants were rested for 30 min. Blood was allowed to clot for 30 min at room temperature before centrifuged at 1,500 g for 15 min at 4 °C. Serum was aliquoted and stored at −80 °C for enzyme-linked immunosorbent assay (ELISA). Serum BDNF levels were measured with a mature BDNF rapid ELISA kit (Biosensis, Thebarton, South Australia) following manufacturer's instructions (85). Serum BDNF levels are used as proxy for BDNF central expression due to the difficulty to measure the levels in the brain. Serum was diluted 1:200 prior use. In brief, 100 µl standard, quality control, blank, and diluted samples were placed into each well in duplicate and incubated at room temperature for 45 min, shaking at 140 rpm. After 5 washes, BDNF detection antibodies were added to each well and incubated for 30 min at 140 rpm before 5 additional washes. Streptavidin-horseradish peroxidase conjugate was added followed by 3,3′,5,5′-tetramethylbenzidine substrate to yield a colored reaction product directly proportional to the concentration of BDNF in samples. A microplate reader (Synergy H1, BioTek, Vermont, USA) was used to read the absorbance at 450 nm and Gen5 software (BioTek) was used to collect and analyze the data.

### Statistical analysis

Statistical analyses were conducted using SPSS 26 (International Business Machines Corp., New Orchard Road, Armonk, NY). The primary objective of this study was to examine within-group differences, with the 6-MinWT as the primary endpoint. A priori power analysis was conducted using the 6-MinWT. At least 10 participants were required to detect within-group differences at a statistical power of 0.80 (one-tailed dependent t-test, expected effect size = 0.9–1.0, α = 0.05). The average of T0-A and T0-B was used for the baseline measurement and was compared to T1 and T2 values. Due to the small sample size and the distribution for some variables not meeting the assumptions of normality, non-parametric analyses were used. The Friedman test with *post hoc* Wilcoxon sign-ranked pairwise comparisons were used for each outcome measure to compare baseline to T1, baseline to T2, and T1 to T2 timepoints. The Benjamini-Hochberg correction procedure using a false discovery rate of 10% was used to interpret level of significance due to multiple *post hoc* pairwise comparisons [e.g., statistical significance when the *p* value is less than Benjamini-Hochberg critical value defined as: (*p* value rank/total number of tests) x false discovery rate] (86, 87). To examine the magnitude of the training effects for those variables that demonstrated statistically significant change the effect size was calculated using the formula (Z/√N) (88). Magnitude of effect size was interpreted as small (<0.30), medium (0.31–0.50), and large (>0.51).

### Role of the funding source

The funders played no role in the design, conduct, or reporting of this study.

## Results

**Figure 1:**
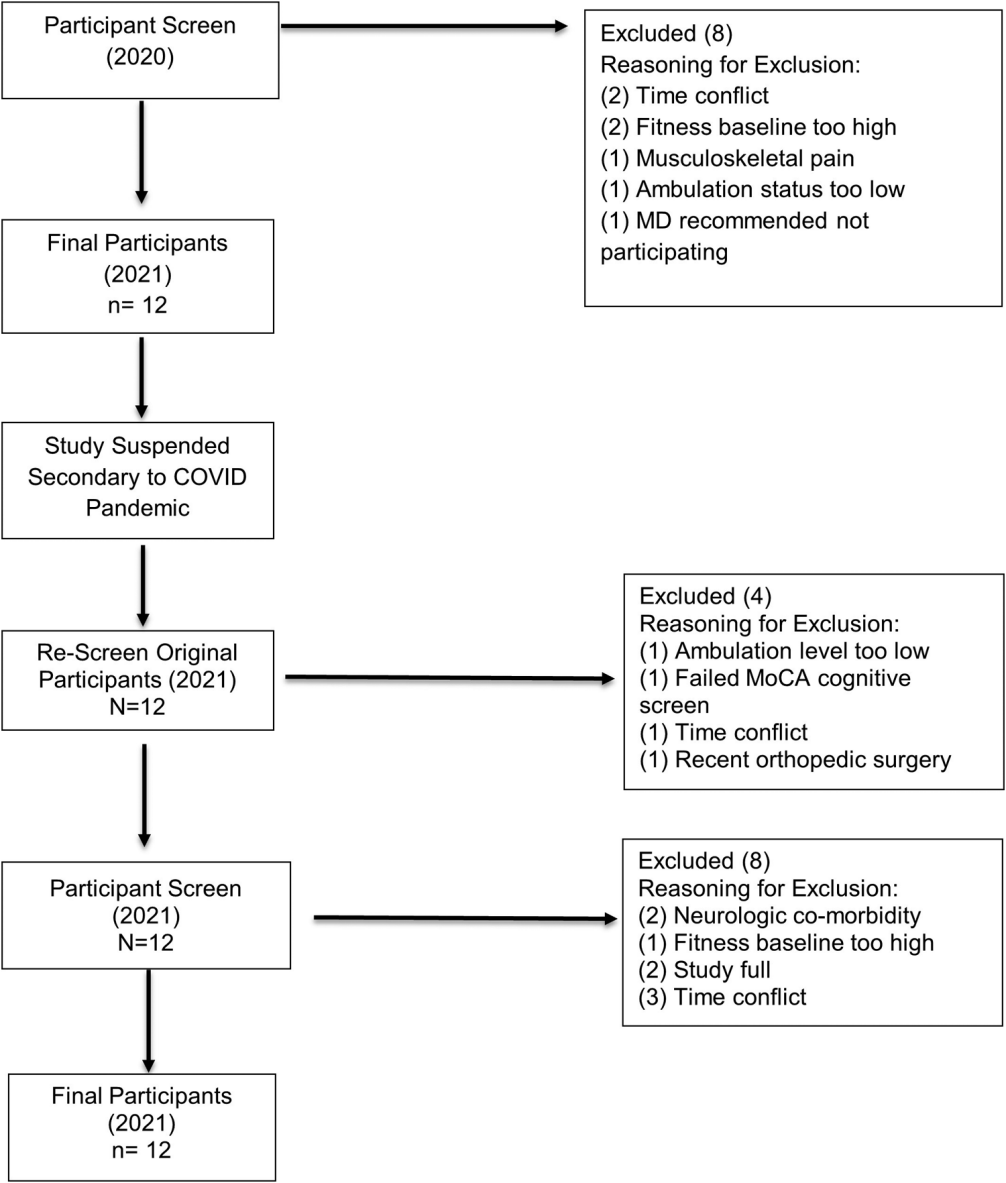
Participant screening flow diagram. Flowchart of selection process and inclusion/exclusion of participants.

Twenty potential participants were originally screened for the study, with 8 participants excluded based on established criteria (Figure 1). Twelve participants qualified for the study and provided informed consent in Winter 2020. The study was then suspended during the COVID pandemic due to the associated restrictions of in-person research activity. Once the suspension was lifted by the University Institutional Review Board, the original 12 participants were rescreened in Spring 2021. Four participants were deemed no longer eligible for the study; therefore, an additional 12 potential subjects were screened. Four of these candidates qualified and consented to participate in the study.

Demographics for the participants in this study are outlined in Table 1. Eight males and four females participated, with mean age 67.17 (SD 9.19; range 46–82) years. Participants were representative of a wide range of disease severity (mean Hoehn / Yahr stage 2.42, range 1–3) as evidenced by MDS-UPDRS scores. PD subtype was analyzed based on MDS-UPDRS scores (89). Participants were classified as Postural and Gait Instability subtype (7 participants), Tremor Dominant subtype (3 participants) and Indeterminate (2 participants). Fifty percent of the sample had a positive fall history at baseline. Five participants had self-reported freezing of gait at baseline. Participants’ baseline activity levels ranged from sedentary (no reported activity- 4 participants), mildly active (30 min of exercise <3 days weekly- 1 participant) to active (>30 min of exercise ≥3 days weekly- 7 participants).

**Table 1:** Participant demographics.

Participant	Age	Sex	Time since PD Onset (yr)	Hoehn and Yahr Stage	UPDRS Total Score	Parkinson's Subtype	Fall history (baseline)	MOCA	Parkinson's Medications	Activity Level	Freezer vs. Non-Freezer
1	60	M	13	3	65	I	**2+**	28	Amantadine, Sinemet, Pramipexole, Inbrija, Entacapone	Sedentary	Fr
2	66	M	0.75	1.5	22	TD	0	30	None	Sedentary	NFr
3	69	M	1	2	26	PIGD	0	29	Sinemet	Active	NFr
4	80	M	1.5	3	30.5	PIGD	**2+**	22^a^	Sinemet	Mildly Active	Fr
5	74	M	5	2	43.5	TD	**2+**	29	Sinemet	Active	Fr
6	68	M	6	3	86.5	PIGD	2	24^a^	Sinemet	Active	Fr
7	64	F	5.25	1.5	26	PIGD	0	26	Sinemet	Active	NFr
8	72	F	5	3	24	PIGD	0	28	Pramipexole, Sinemet	Sedentary	NFr
9	62	M	3.75	2	41	I	1	26	Sinemet	Active	NFr
10	46	F	3.75	2	47.5	PIGD	0	26	Sinemet, Mirapex	Active	NFr
11	82	F	3.75	3	47	PIGD	1	27	Sinemet, Carbidopa	Sedentary	NFr
12	63	M	5	2	58	TD	0	28	Sinemet	Active	Fr

^a^ = suggests mild cognitive impairment; UPDRS, Unified Parkinson's Disease Rating Scale; Sedentary, no reported activity prior to study; Mildly active, 30 min of exercise less than three days weekly; Active, >30 min of exercise three or more days weekly; M, Male, F, Female, Bolded fall data, injurious fall; Fr, Freezer; NFr, Non-Freezer; TD, PD Subtype (79): Tremor Dominant; PIGD, Postural Instability and Gait Disturbance; I, Indeterminate.

### Training outcomes

#### Daily physical activity

**Figure 2:**
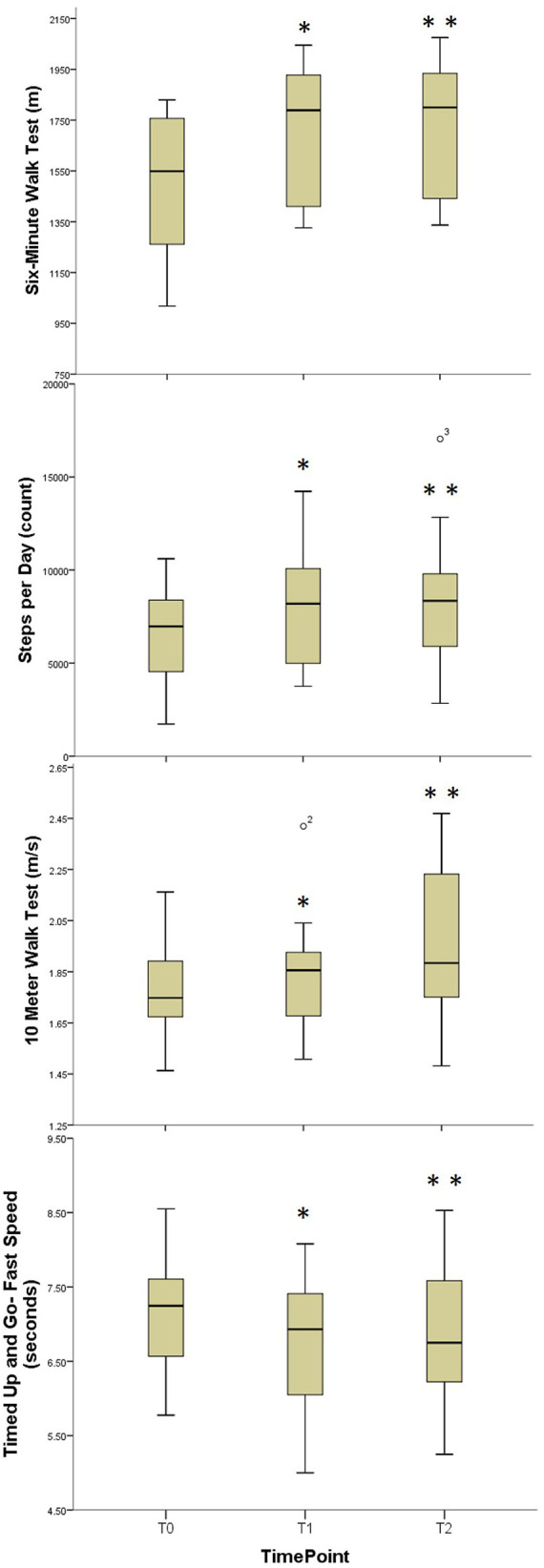
Comparative boxplots for walking function outcome measures. Boxplots for Aerobic Capacity, Daily Physical Activity, Gait Speed, and Functional Mobility; T0, Baseline; T1, Immediate Post-Training; T3, Three Month Follow-Up; *, Statistically significant difference T0-T1; **, Statistically significant difference T1-T2.

Significant improvements in average number of steps per day were observed following the supervised training phase and were retained at long term follow-up [*χ*^2 ^= 12.5, *p* = 0.002, Median improvement T0-T1 = 1,226.7 (268.2, 2726.7) steps per day, effect size = 0.82; Median improvement T0-T2 = 1,368.7 (561.0, 3609.0) steps per day, effect size = 0.82] (Table 2, Figure 2) All but two subjects improved by at least 500 steps per day, with three subjects improving by more than 4,000 steps per day.

**Table 2:** Selected nordic walking training outcomes.

Outcome Measure	Baseline (T_0_) Mdn (IQR)	Post-Training (T_1_) Mdn (IQR)	Follow-up (T_2_) Mdn (IQR)	Change T_0_ -T_1_ Mdn (IQR)	Change T_0_ – T_2_ Mdn (IQR)
6MWT (m)	473.6	547.0	550.5	66.8^a^	73.0^a^
	(376.5,539.0)	(424.8, 589.8)	(431.9, 593.3)	(42.4,93.7)	(45.0,92.1)
Steps per Day (count)	6976.1	8187.7	8349.0	1226.7^a^	1368.7^a^
	(3840.6, 8494.3)	(4427.5, 10,183.4)	(5581.6,10083.1)	(268.2, 2726.7)	(561.0, 3609.0)
10 mWT comfortable speed (m/s)	1.29	1.33	1.38	0.05	0.10^a^
	(1.14,1.37)	(1.26, 1.44)	(1.26, 1.49)	(0.01, 0.11)	(0.00, 0.14)
10 mWT fast speed (m/s)	1.75	1.86	1.88	0.07	0.16^a^
	(1.66, 1.92)	(1.65, 1.93)	(1.73, 2.29)	(−0.01, 0.17)	(0.07, 0.26)
TUG-fast speed (sec)	7.3	6.9	6.8	0.4^a^	0.3^a^
	(6.5, 7.6)	(6.0, 7.5)	(6.2, 7.6)	(0.1, 0.7)	(0.0, 0.6)
Stride Length (left) comfortable speed (m)	1.40	1.47	1.55	0.07	0.12^a^
	(1.26, 1.56)	(1.33, 1.61)	(1.41, 1.63)	(−0.04, 0.18)	(0.00, 0.16)
Stride Length (right) comfortable speed (m)	1.39	1.47	1.52	0.07	0.14
	(1.26, 1.56)	(1.32, 1.60)	(1.41, 1.61)	(−0.05, 0.19)	(−0.00, 0.17)
Nonmotor Symptom	68.5	74.5	62.5	14.5	17.0
Scale (score)	(51.3, 115.0)	(50.0, 84.5)	(39.5, 91.0)	(−14.8, 33.3)	(−7.8, 30.8)
MDS-UPDRS Total	42.3	25.0	26.0	9.8	11.25
Score	(26.0, 55.4)	(20.5, 43.5)	(21.3, 43.3)	(4.5,18.4)^a^	(3.0, 15.9)
MDS-UPDRS Motor Score	20.3	10.0	11.0	8.0	6.3^a^
	(9.3, 28.8)	(7.25, 16.5)	(8.0, 18.0)	(3.0,12.6)	(2.0,10.0)
PFS Score	41.0	43.0	49.0	−2.0	−.50
	(23.5, 54.0)	(26.0, 58.3)	(23.0, 59.5)	(−11.8, 2.0)	(−11.5, 1.25)
FOG Score	0.0	0.0	0.0	0.0	0.0
	(0.0, 14.5)	(0.0, 11.3)	(0.0, 11.3)	(0.0, 2.9)	(0.0, 0.0)
Serum BDNF (ng/ml)	34.8	32.3	38.7	−2.1	6.8
	(27.7, 37.3)	(27.3, 36.8)	(31.5, 55.8)	(−6.70, 3.71)	(0.59, 17.8)

^a^ Statistically significant difference following Benjamini-Hochberg correction. Abbreviations: 6 MWT, 6-minute walk test; 10 MWT, 10-meter walk test; TUG, timed up and go; NMS, Non-motor Symptom Scale; MDS-UPDRS, Movement Disorders Society-revised Unified Parkinson's Disease Rating Scale; Mdn, median; IQR, interquartile range.

#### Gait measures- 6-minWT, 10 mWT, spatiotemporalParameters

Significant improvements in 6-MinWT distance were found following the supervised training phase and were retained at long term follow-up [*χ*^2 ^= 19.5, *p* < 0.001, Median improvement T0-T1 = 66.8 m (42.4, 93.7), effect size = 0.88; Median (IQR) improvement T0-T2 = 73 m (45.0, 92.1), effect size = 1.01] (Table 2, Figure 2). All subjects demonstrated improvement in 6-MinWT distance.

Significant improvements in the comfortable-pace walking speed based on the 10 mWT were observed at long-term follow-up [*χ*^2 ^= 6.78, *p* = 0.034, Median improvement T0-T2 = 0.10 (0, 0.14) m/s, effect size = 0.57]. Similarly, significant improvements in fast-pace walking speed were observed at long-term follow-up [*χ*^2 ^= 10.09, *p* = 0.006, Median improvement T0-T2 = 0.16 (0.07, 0.26) m/s, effect size = 0.88] (Table 2, Figure 2).

The primary spatiotemporal parameters measured using the G-Walk included stride variability, stride length, and step length symmetry. In general, the study was significantly under powered regarding spatiotemporal parameters with achieved power less than 0.30 for stride variability and step length symmetry. Improvements in right and left stride length during comfortable and fast gait of approximately 0.15 meters were noted, but only approached significance (except left comfortable stride length which was statistically significant) and were unpowered as well with achieved power less than 0.50.

#### Functional mobility measures

Small but statistically significant improvements in comfortable-pace TUG were observed at follow-up [*χ*^2 ^= 6.5, *p* < 0.039, Median improvement T0-T2 = 0.62 (0.10, 0.82) seconds, effect size = 0.63]. Fast-paced TUG demonstrated small but statistically significant improvements immediately following supervised training that were retained at long term follow-up [*χ*^2 ^= 10.5, *p* = 0.005, Median improvement T0-T1 = 0.38 (0.10, 0.70) seconds, effect size = 0.71; Median improvement T0-T2 = 0.26 (0, 0.6) seconds, effect size = 0.68] (Table 2, Figure 2). There were no changes noted in dual-task motor or cognitive-TUG performance at either comfortable or fast pace.

#### Disease severity, motor and non-motor symptoms

**Figure 3:**
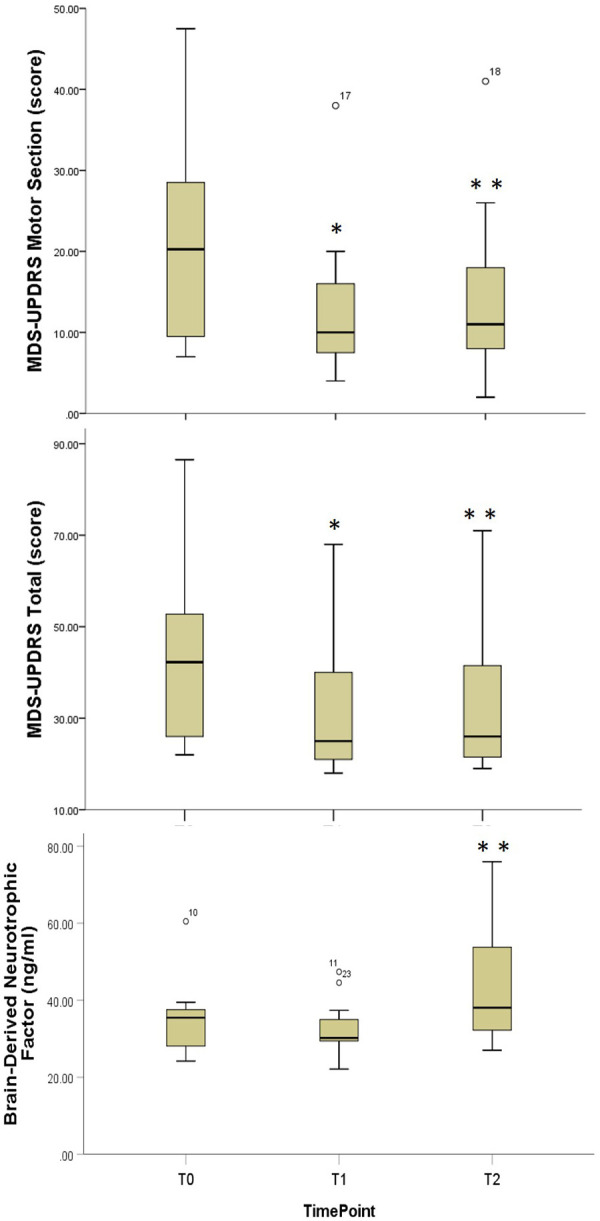
Comparative boxplots for disease severity and exercise biomarker outcome measures. Boxplots for Movement Disorders Society-Revised Unified Parkinson's Disease Rating Scale; T0, Baseline; T1, Immediate Post-Training; T3, Three Month Follow-Up; *, Statistically significant difference T0-T1; **, Statistically significant difference T1-T2.

Significant improvements in both MDS-UPDRS total and MDS-UPDRS Motor (III) scores were observed following the supervised training phase [*χ*^2 ^= 13.6, *p* = 0.001, Median difference T0-T1 = 9.8 (4.5, 18.4) points, effect size = 1.03; *χ*^2 ^= 12.7, *p* = 0.002, Median improvement T0-T1 = 8.0 (3.0, 12.6) points, effect size = 0.97, respectively] (Table 2, Figure 3). The MDS-UPDRS Motor scores were maintained at the long-term follow-up. [Median difference T0-T2 = 6.3 (2.0, 10.0) points, effect size = 0.71] (Table 2, Figure 3). There were no significant changes seen in FOG, and PFS scores. Although the NMS-total score did not show statistically significant change scores, 9 out of 12 participants had a decrease in total MDS-NMS score (mean = 34.77, range = 11–110) with reduced nonmotor symptoms, whereas 3 out of the 12 participants had an increase in total MDS-NMS (mean = 37.33, range = 13–56).

#### Serum brain-derived neurotrophic factor

Serum BDNF at 3-month follow-up was statistically significantly greater than both baseline and post-supervised training phase [*χ*2 = 12.2, *p* = 0.002, Median improvement T0-T2 = 6.8 (0.6, 16.7) ng/mL, effect size = 0.75; Median improvement T1-T2 = 10.0 (5.3, 17.8) ng/mL, effect size = 0.73, respectively] (Table 2, Figure 3).

### Safety and feasibility

#### Adverse events/ safety of NW exercise

There were no major adverse events, including no falls during supervised or unsupervised NW training exercise and no major cardiovascular events.

#### Adherence to NW exercise

Adherence to the supervised and independent phases of the training protocol was good (Tables 3, 4). Individual participants' attendance rate to supervised training sessions ranged from 79%–100%, with an average of 95% attendance rate. Based on review of activity logs, nine out of the twelve participants met the target home NW exercise goal for at least five out of six weeks during the supervised NW training phase. During the Independent Phase, the target goal was engagement in NW exercise at least three times weekly. Fifty eight percent of the participants met the target goal in months one and two, and 66% of participants met the goal in month three. It is notable that ten of the twelve participants met this goal for the majority of weeks during this Independent Phase, whereas two participants had relatively lower adherence.

**Table 3:** Supervised training phase: NW exercise training distances.

Participant	Week 1 with trainer (Avg miles per session)	Week 1 Total Miles	Week 3 with trainer (Avg miles per session)	Week 3 Total Miles	Week 6 with trainer (Avg miles per session)	Week 6 Total Miles	# of Weeks Unsupervised NW Goal Met
1	1.92	5.75	2.48	10.05	2.36	5.55	5 out 6
	(0.71)		(0.25)		(0.22)		
2	1.60	7.2	2.03	7.23	2.02	6.86	2 out of 6
	(0.48)		(0.01)		(0.14)		
3	1.78	13.9	1.89	12.47	2.27	10.94	6 out of 6
	(0.32)		(0.06)		(0.42)		
4	1.93	4.25	1.89	6.38	1.83	10.51	6 out of 6
	(0.45)		(0.18)		(0.47)		
5	1.94	6.08	2.26	6.52	2.6	7.77	6 out of 6
	(0.33)		(0.08)		(0.14)		
6	1.08	6.48	1.5	6.49	1.64	8.47	6 out of 6
	(0.33)		(0.10)		(0.39)		
7	1.17	4.28	1.54	6.02	1.59	4.92	2^a^ out of 6
	(0.28)		(0.12)		(0.05)		
8	1.40	6.69	1.74	6.23	1.95	5.9	5 out of 6
	(0.34)		(0.25)		(0.07)		
9	1.82	6.76	2.21	8.35	2.41	6.91	4 out of 6
	(0.48)		(0.42)		(0.35)		
10	1.63	8.28	2.03	10.15	2.19	6.94	6 out of 6
	(0.49)		(0.07)		(0.13)		
11	0.77	3.16	0.83	7.32	1.4	7.05	6 out of 6
	(0.21)		(0.69)		(0.14)		
12	1.80	9.26	2.14	11.76	2.08	9.67	6 out of 6
	(0.25)		(0.07)		(0.09)		
Aggregate Data	1.57	6.84	1.87	8.25	2.03	7.62	75% met target goal^a^
	(0.38)	(2.81)	(0.44)	(2.29)	(0.36)	(1.92)	

NW, Nordic Walking.

^a^ Target goal was 5 out of 6 weeks.

**Table 4:** Independent training phase: NW exercise engagement monthly average.

Participant	Month 1 (Avg. miles per session) (Avg. min. per session)	Month 1 (# of Weeks Independent NW Goal Met)	Month 2 (Avg. miles per session) (Avg. min. per session)	Month 2 (# of Weeks Independent NW Goal Met)	Month 3 (Avg. miles per session) (Avg. min. per session)	Month 3 (# of Weeks Independent NW Goal Met)
1	2.1 (1.2)	5 out of 6	1.8 (1.1)	3 out of 4	1.7 (1.1)	4 out of 4
					54.4 (22.1)		
	58.4 (24.9)		48.1 (18.1)				
2	4.6 (1.0)	5 out of 6	5.7 (0.6)	2 out of 4	4.8 (0.9)	1 out of 4
			93.8 (10.2)		73.8 (21.7)		
	79.1 (18.1)						
3	2.5 (0.8)	5 out of 6	2.3 (0.4)	4 out of 4	2.4 (1.0)	4 out of 4
					48.7 (21.6)		
	48.8 (16.7)						
4	2.2 (1.0)	5 out of 6	2.4 (0.9)	4 out of 4	2.5 (0.6)	4 out of 4
					53.9 (8.4)		
			48.6 (14.1)				
	45.5 (10.1)						
5	2.8 (0.5)	6 out of 6	2.9 (0.4)	4 out of 4	3.0 (0.5)	4 out of 4
	54.0 (9.5)		53.8 (8.1)		54.3 (8.5)		
6	1.7 (0.5)	6 out of 6	1.7 (0.3)	4 out of 4	1.8 (0.5)	4 out of 4
	25.5 (10.8)						
			29.3 (6.1)				
					30.1 (6.0)		
7	1.6 (0.6)	6 out of 6	1.3 (0.5)	3 out of 4	1.7 (0.4)	1 out of 4
	34.3 (13.3)		28.5 (9.5)		39.6 (8.9)		
8	not reported 20^a^	6 out of 6	not reported 20^a^	4 out of 4	not reported 20^a^	4 out of 4
9	2.0 (0.3)	6 out of 6	1.6 (0.3)	2 out of 4	2.0 (0.2)	3 out of 4
	39.8 (8.0)		32.0 (6.4)		36.7 (4.7)		
10	1.8 (0.6)	6 out of 6	1.9 (0.2)	4 out of 4	1.7 (0.4)	3 out of 4
	36.7 (11.4)		38.6 (5.3)		41.7 (6.8)		
11	0.9 (0.3)	5 out of 6	0.8 (0.4)	2 out of 4	1.0 (0.6)	4 out of 4
			20.2 (11.2)		27.0 (14.0)		
	24.9 (9.7)						
12	1.8 (0.6)	6 out of 6	1.9 (0.5)	4 out of 4	1.7 (0.5)	4 out of 4
			47.1 (10.8)		(44.4) (10.7)		
	44.7 (12.8)						
Aggregate Data	2.2 (1.0)	58% met target goal	2.2 (1.3)	58% met target goal	2.2 (1.0)	66% met target goal
					43.7 (14.6)		
			42.1 (19.9)				
	42.6 (16.5)						

Month 1, 6wks; Month 2, 4wks; Month 3, 4wks; NW, Nordic Walking; Avg, average; averages listed as mean (standard deviation); Independent, Nordic walking without a trainer; % met target goal, goal of at least 3 days per week of NW was met every week.

^a^ verbally estimated report.

#### Feasibility and sustainability

Throughout the study, participants achieved the intensity goal set for NW training, exercising at a moderate intensity, with high intensity achieved during interval training. With regards to walking endurance demands, 10 of 12 participants met the goal of continuous NW exercise for 45 min. Feasibility throughout the 3-month Independent Phase is reflected by the overall NW exercise engagement in this cohort (Table 4). As noted above the majority of participants met the target goal of engaging in NW exercise independently three times weekly. Average miles walked and average minutes walked per exercise session (approximately 2.2 ± 1.0 miles) remained consistent across the 3 months of independent training.

## Discussion

The present study investigated the immediate and long-term effects of NW exercise on walking function, motor/non-motor PD symptoms and exercise-related biomarkers in persons with mild to moderate idiopathic PD. This study was designed to address several important gaps in previous research, including high variability in NW dosing and implementation, a lack of data on feasibility, a focus on immediate training effects, and a lack of long-term follow-up. Most notably, this proof-of-concept study was designed to assess if (1) changes in clinical outcome measures were associated with changes in serum BDNF and (2) if there was a long-term, sustained engagement in NW aerobic exercise and retention of training-induced improvements across a wide variety of endpoints. Following engagement in supervised NW exercise the participants in this sample demonstrated significant improvements in walking endurance, average daily steps (ambulatory activity), gait speed, and PD motor symptoms. Serum BDNF levels increased from baseline to follow-up, paralleling these changes in clinical measures. Furthermore, participants safely sustained their engagement in NW exercise for 3 months following training with the improvements in clinical measures maintained.

### Training effects on daily physical activity

Regarding daily physical activity, the participants in this study showed significant short-term increases in average daily step counts post-training that were retained at long-term follow-up. Previous research has shown that decreased daily ambulatory activity and bouts of sustained walking can lead to a higher risk of gait and functional decline in those with PD (13, 14). Multiple longitudinal studies provide evidence that an increased level of physical activity and greater step count in those with PD is correlated with neuroprotective effect for slower disease progression, reduced disability, preservation of walking function, and improved QOL as compared to those who are less physically active and take less steps per day (21, 22, 90, 91). Given that the present study only tracked total daily step counts, it is not clear whether the improvements in daily activity were solely due to the NW exercise sessions or whether the improvements in functional walking endurance translated into an overall increase of daily walking activity.

### Training effects on gait function

Walking endurance (6-MinWT) improved after the supervised training phase and was sustained following the 3-month independent exercise phase. The magnitude of improvement of approximately 70 m in 6-MinWT found in this study is clinically meaningful change and is nearly identical to that reported by Cugusi et al. (54). The results of the present study uniquely add to the literature by demonstrating that this benefit can be retained following 3 months of independent NW exercise.

Although gains in 6-MinWT distance could be the result of improvements in gait quality/walking function, it is reasonable to assume that at least some of this change was due to improvement in aerobic capacity. The clinical importance of improved aerobic capacity in people with PD cannot be understated given that scientific studies in both animal and clinical research provide evidence that moderate to high intensity aerobic exercise enhances brain health and supports neuroplasticity in PD (10, 22, 23). NW exercise is a moderate intensity exercise program that produces improved aerobic function (41–43), which may have neuroprotective effects in persons with PD if there is sustained engagement in exercise. Preliminary analysis of exercise related biomarkers in this study revealed that there was an increase in serum BDNF levels over the 5-month study intervention, which may support brain health and neuroplastic processes (22, 23, 31, 32).

Significant and sustained increases were observed in gait speed from baseline to 3-month follow-up for both comfortable and fast walking speeds. The observed increase in comfortable gait speed of 0.10 m/s is clinically meaningful as it falls within the estimated minimum clinically important difference (MCID) of.05 m/s to.22 m/s (92). Only one previous study examined retention of training effects, and our findings were consistent with those of VanEijkeren (48) demonstrating retention of improved gait speed. Sustained NW exercise as seen over this 5-month period may improve walking efficiency and speed (49), thereby optimizing gait function and reducing gait decline commonly seen in PD.

No consistent or significant quantitative changes in stride length, stride variability, or step length symmetry were found as measured by the G-Walk. Improvements of stride length in both comfortable and fast 10 mWT were clinically relevant (0.15 m) but only approached statistical significance and may have been unpowered. Our findings regarding improvement in stride length following NW training agree with previous research, supporting that NW exercise addresses the step hypokinesia seen in PD gait (7, 40, 47, 49). Given that improvements in gait quality were anecdotally observed across participants (upright posture, increased step length and arm-swing, gait rhythm), we hypothesize that the lack of quantitative improvement in spatiotemporal gait parameters may be due to the short distance (measured over only 8 meters during 10mWT) that was used for G-Walk sampling. As previously noted, all participants in the present study demonstrated significant improvements in functional walking distance and sustained walking speed as measured by the 6-MinWT. As aerobic capacity was not measured using cardiopulmonary exercise testing, it is not known whether the improvement in 6-MinWT distance was due to improvements in aerobic capacity, improvements in temporal-spatial gait parameters, or a combination of both. Future research should measure spatiotemporal gait parameters during the 6-MinWT to elucidate the ways in which NW exercise training impacts gait quality over periods of sustained walking.

Five participants reported freezing of gait at baseline based on FOG. There were no statistically significant changes in FOG questionnaire scores post-training or at follow-up. However, researchers qualitatively noted that there were reduced observed freezing episodes during the post-training and follow-up assessments. Additionally, there were only minimal episodes of freezing observed by the trainers during the supervised training phase. Freezing of gait characteristics did not affect participants' ability to safely participate in the NW exercise, and those individuals with freezing made comparable gains in their gait function as those non-freezers.

### Training effects on Parkinson's motor and nonmotor symptoms

The MDS-UPDRS, which reflects the severity of motor and nonmotor PD-related deficits and the effects of disease on activities of daily living, is the gold standard for assessment of efficacy of interventions on disease severity. Following engagement in NW exercise the participants in this study demonstrated significant short-term and long-term improvement in MDS-UPDRS total and motor scores, which is consistent with findings reported in meta-analysis by Bombieri et al. (44) who found a standardized mean difference/overall effect size of 0.64 for the MDS-UPDRS motor scores across studies examining the short-term effects of NW. The larger effect size in MDS-UPDRS-motor scores in our study (0.97 for T0-T1 and 0.71 for T0-T2) may be attributed to our individualized and progressive training approach. The MDS-UPDRS-motor change score reported in this study surpassed the threshold for the MCID of 3.25 (78). The long-term changes (T0-T2) in total MDS-UPDRS scores [11.25(12.88)] found in this study were greater than those changes reported (4.1) by Krishnamurthi et al. (93) and Ebersbasch et al. (56) [0.58(3.17)] after 16 weeks. Over a 5-month period of engagement in NW exercise, there was no evidence of decline in PD symptoms based on participants' MDS-UPDRS total and motor scores, with the majority of participants demonstrating improved scores.

Although the MDS-NMS did not show an overall statistically significant difference in nonmotor symptoms post-training or at the 3-month follow-up, seventy-five percent of individuals in this study had an improvement in their nonmotor symptoms (mean = 34.77, range = 11–110). The improvement trends were in the sleep, pain and apathy domains. Twenty-five percent of the sample had a reported worsening of nonmotor symptoms (mean = 37.33, range = 13–56); notably they reported worsening in cognition and anxiety domains. We suspect that the decline in MDS-NMS scores in these 3 participants may have been due to the complex, multiple factors that can affect participant's self-ratings at any timepoint, especially ratings on mood dependent on current life circumstances. These 3 participants had reported significant psychosocial or life stressors at the T1 and T2 assessments. The present study was unique in using the Movement Disorder Society recommended PD-specific NMS, which utilizes a self-report rating of severity and frequency of nonmotor symptoms, and their impact on daily function. This tool may have measured the non-motor effects of NW differently than stand-alone measures of cognitive and depression as reported by Passos-Monteiro et al. (57).

Regarding fatigue, there were no notable improvements in PFS scores. The reason for this is unclear. Since this measure assesses mainly the physical aspects of fatigue, this finding could be attributed to the increased physical demands during our study, increasing the overall amount of fatigue perceived. Fatigue is a complex construct with multifaceted possible contributing elements, including central fatigue, mental fatigue, physical fatigue, and peripheral fatigue (94). Therefore, fatigue is a challenging nonmotor symptom to measure and the PFS was limited to examining mainly the effects of physical fatigue on daily function.

### Training effects on serum brain-derived neurotrophic factor

Physical exercise is known to increase BDNF which regulates synaptic transmission and activity-based neuroplasticity. In Parkinsonian rodent models, exercise training increased BDNF in the striatum, mitigated dopaminergic neurotoxin, and induced new dopaminergic terminal sprouting, suggesting neuroprotection effects (95, 96). A recent study also demonstrated a correlation between increased BDNF and striatal dopamine release after a 30-day exercise training program in mice (97). In humans, various types of intensive exercise such as treadmill training, cycling, and balance training significantly increased serum BDNF levels in people with PD following 4 to 12 weeks of exercise training (33, 98, 99). To the best of our knowledge, we are the first to measure the effect of a longer term/20-week NW exercise program on BDNF in people with PD. Although the underlying molecular mechanisms of exercise-induced increase in serum BDNF remain unknown, the elevation of BDNF levels may be due to the increase BDNF production in the brain (33, 98). Studies have shown decreased BDNF gene expression in postmortem PD brains and that decreased serum BDNF levels were positively correlated with striatal dopaminergic degeneration in people with PD (28, 100). Decreased BDNF levels in serum may contribute to the inhibition of dopamine production and increase dopaminergic neuron degeneration, leading to movement impairment and other deficits (101). Thus, increased serum BDNF levels may be linked to the motor and non-motor improvements in the present study, suggesting long-term NW exercise might have neuroprotective effects in people with PD.

### Safety and feasibility of supervised and independent NW exercise

There were no major adverse events during NW supervised or independent exercise sessions, which provides support that this intervention is a safe mode of exercise for persons with PD with a range of disease severity. The 1:2 trainer to participant ratio provided a safe environment for supervised NW exercise in individuals with a wide range of gait and balance dysfunction. Once participants developed NW skill and balance confidence during supervised training, they were able to safely engage in NW independently. Several participants had a reported history of 2 or more falls at baseline, and subsequently, had reduced the number of falls during the study's intervention phase. No falls occurred during NW training, either supervised or independent exercise sessions.

Nordic Walking exercise, delivered in an individualized and progressive manner was a feasible and beneficial mode of exercise for individuals with mild to moderate stage PD. Participants were able to achieve the training goals of this supervised NW exercise program, namely good adherence to exercising 3 times weekly, exercised at moderate intensity with intermittent high intensity intervals; and engaged in 45 min of continuous NW with short standing breaks. Training with a small group created a sense of community, working towards the common goals of improving their walking function and fitness.

### Training individualization and progression

A unique aspect of this study is the inclusion of a detailed description of how the NW intervention was specifically implemented, individualized, and progressed. Training individualization was provided to each participant through instructional cues based on their gait impairments and NW skill level. Common gait impairments addressed among the participants during NW included increasing arm swing, stride length, and promoting upright posture. Ensuring proper NW technique is critical for addressing the hypokinesia element of gait dysfunction in persons with PD. As individuals mastered basic NW skill trainers systematically added training progressions, such as speed/effort intervals, hills, and varied terrains.

### Sustainability of independent NW exercise

During the supervised training phase participants developed foundational NW skills and fitness for walking endurance that supported the independent exercise phase. Engagement in NW exercise was sustained following the supervised training phase as evidenced by the data collected from the weekly activity logs during the 3-month independent NW exercise phase. Overall, participants maintained their distance and duration of NW exercise during all three months (Table 4). This sustained exercise engagement facilitated maintenance of gains in the clinical measures and for some participants further improvements in aerobic, motor, and walking function. The significant increases in serum BDNF levels from baseline to T2 for participants in this study was associated with the sustained engagement in 5 months of NW exercise, which may lend support to the possible contribution of higher intensity exercise to facilitate neuroplasticity and neuroprotective benefits.

### Limitations and future research

Due to the interprofessional nature of this proof-of-concept study and the costs associated with serial laboratory biomarker analysis, this was a pilot study conducted with small internal grant funding with the aim of obtaining data to support a larger scale funded study. Therefore, there are several limitations to note in the present study. One of which is the lack of control group and so causation cannot be assumed. Due to our small sample size, generalizability to the larger PD population may be limited; however, our participants represented a range of PD severity and gait function. Those researchers who conducted the assessment measures were not involved in NW intervention, however they were not blinded to timepoints, except for the examiner who scored MDS-UPDRS who was blinded. Although examiners were not blinded, the gait outcome measures were all timed measures with no subjective scoring and NMS and PFS were self-report questionnaires. There were also limitations found with use of the Fitbit device. At the initial setup of the participants' accounts, weight, height, and step length were not added to their individual profiles. This data omission resulted in less accuracy in reports on ambulation distance and lack of accurate data on time spent in active minutes per day. We did not evaluate functional balance measures as an outcome in this study and only monitored fall reports; therefore, this limited out ability to analyze the direct effects of this intervention on fall risk.

Given our results from this feasibility study, future research studies should employ randomized controlled trials with a larger sample size and active control group to further enable generalization and give a comparative analysis with other exercise-based interventions. Additionally, a balance outcome measure should be included and investigate how NW affects balance function and fall risk. Longitudinal studies are needed to further explore the effects of NW on exercise-related biomarkers to shed light on the possible neuroprotective effects of this sustained exercise in persons with PD.

## Conclusions

This study uniquely addressed significant gaps in the literature, including high variability in NW dosing and implementation, a lack of data on feasibility, a focus on immediate training effects, a lack of long-term follow-up or examination of independent NW exercise following training, and most importantly, whether changes in exercise biomarkers, namely serum BNDF levels, are observed in parallel with the changes observed in clinical outcome measures. Following a supervised six-week NW exercise intervention, which was delivered in an individualized and progressive manner, there was sustained, independent three-month engagement in NW with retention of significant improvements in walking endurance, average daily steps (ambulatory activity), gait speed, and PD motor symptoms. Additionally, improvements in non-motor PD symptoms were reported for 9 out of the 12 participants based on the NMS scores. An important finding is that along with these clinical improvements, participants had a significant increase in serum BDNF levels. NW exercise was a safe, feasible, and sustainable mode of task-specific aerobic exercise training for participants in this study who had varied disease severity and gait dysfunction. Therefore, further exploration of whether ongoing NW exercise engagement ameliorates gait decline and provides neuroprotective effects mediated by neurotrophic factors in persons with PD is warranted.

## Data Availability

The raw data supporting the conclusions of this article will be made available by the authors, without undue reservation.
